# Incidental Detection of Situs Inversus Totalis During Preoperative Evaluation for Thyroid Cancer: A Case Report

**DOI:** 10.7759/cureus.91991

**Published:** 2025-09-10

**Authors:** Carlos E Solórzano, Osvani Leyva Matos

**Affiliations:** 1 Internal Medicine, National Autonomous University of Honduras, Tegucigalpa, HND; 2 Internal Medicine, American University of the Caribbean School of Medicine, Philipsburg, SXM

**Keywords:** congenital anatomical variant, papillary thyroid carcinoma, preoperative diagnosis, pre-surgical planning, situs-inversus-totalis

## Abstract

Situs inversus totalis (SIT) is an uncommon congenital condition in which the thoracic and abdominal organs are arranged in a mirror-image orientation. While often asymptomatic, undiagnosed SIT can complicate clinical evaluation, imaging interpretation, and surgical planning.

We present the case of a 60-year-old woman with a palpable neck mass diagnosed as papillary thyroid carcinoma. During the preoperative assessment, a physical examination revealed right-sided heart sounds, and an electrocardiogram showed a right axis deviation with inverted P waves. Chest radiography and abdominal imaging confirmed complete thoracoabdominal organ reversal consistent with SIT, without associated cardiac anomalies. The patient underwent a successful total thyroidectomy with no intraoperative complications. Histopathology revealed multifocal disease with capsular invasion. Postoperative recovery was uneventful, and follow-up care included thyroid hormone replacement and surveillance imaging.

This case highlights the rare coexistence of SIT and papillary thyroid carcinoma, underscoring the importance of recognizing SIT in surgical and anesthetic planning. Routine preoperative imaging plays a critical role in identifying anatomic variants, preventing misinterpretation of clinical findings, and minimizing perioperative risk. While no causal link between SIT and thyroid carcinoma can be inferred from a single case, the report adds to the very limited literature and emphasizes the need for vigilance in both diagnostic evaluation and operative management of patients with SIT.

## Introduction

Situs inversus totalis (SIT) is a rare congenital anomaly in which the thoracic and abdominal viscera are arranged as a mirror image of normal anatomy, occurring in approximately 1 in 10,000 live births [1]. The condition results from disrupted left-right signaling during early embryogenesis and is associated with variants in ciliary and laterality genes [1]. While many individuals with SIT remain asymptomatic, its presence has important clinical implications. For example, acute appendicitis may present with left lower quadrant pain rather than the typical right-sided location, and dextrocardia may cause electrocardiograms to mimic ischemic or conduction abnormalities if lead placement is not adjusted [2]. Such variations highlight how unrecognized SIT can confound physical examination findings, lead to diagnostic delays, and increase perioperative risk [2].

From an epidemiological standpoint, SIT is usually inherited in an autosomal recessive pattern and may occur either in isolation or as part of syndromic conditions. Up to 50% of cases are associated with Kartagener’s syndrome, a subset of primary ciliary dyskinesia characterized by the triad of situs inversus, chronic sinusitis, and bronchiectasis [3]. Recognition of these associations is important, as respiratory comorbidities may further increase anesthetic and perioperative risk. A structured imaging approach, beginning with chest radiography and abdominal ultrasonography and extending to cross-sectional modalities when indicated, can accurately delineate organ position, guide patient positioning, and enable anesthesiologists to plan vascular access [2,4]. Electrocardiographic indicators such as right-axis deviation and inverted P waves in leads I and aVL may suggest SIT but can be overlooked if the diagnosis is not considered [2].

Although SIT has been reported in conjunction with various intra-abdominal malignancies, incidental identification during evaluation for thyroidectomy is exceptionally uncommon [5]. To date, only a few cases describing the coexistence of SIT and papillary thyroid carcinoma have been reported, underscoring the rarity of this association [5]. Concurrently, the global incidence of papillary thyroid carcinoma continues to rise, driven by expanded imaging protocols and enhanced surveillance [6]. Early recognition of anatomic variants such as SIT is therefore essential to prevent diagnostic errors, minimize operative complications, and facilitate effective multidisciplinary communication.

We describe the case of a 60-year-old female with papillary thyroid carcinoma in whom SIT was first detected during routine preoperative assessment. This case highlights both the diagnostic challenges and perioperative considerations associated with SIT and contributes to the very limited literature on its coexistence with thyroid malignancy.

## Case presentation

**Figure 1:**
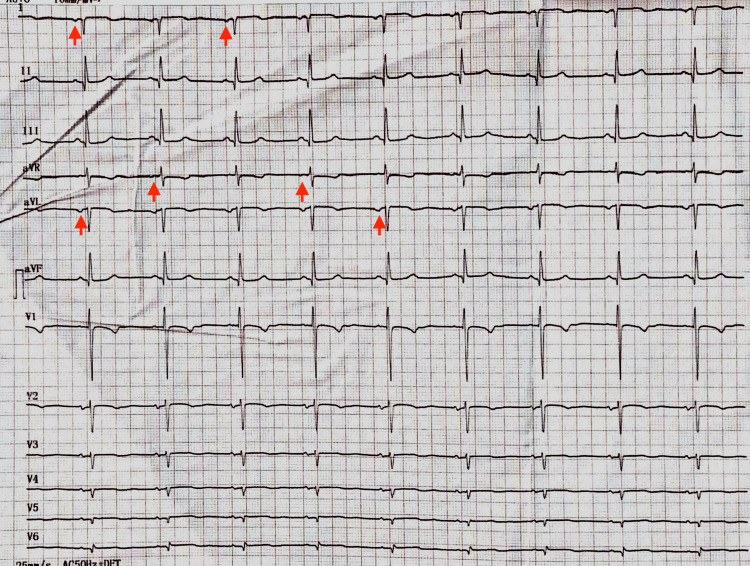
Electrocardiogram (ECG). ECG revealing right axis deviation and inverted P waves on leads I, avL, and avR (red arrows).

A 60-year-old female, from southwestern Honduras, with a past medical history of type 2 diabetes mellitus and hypertension, presented with a palpable, non-tender cervical mass. Ultrasound examination revealed a calcified lesion located in the superior pole of the left thyroid lobe, measuring 27.2 × 20.0 mm. Thyroid function tests were within normal limits (thyroid-stimulating hormone (TSH) 0.5 μIU/mL, thyroxine (T4) 4.6 μg/dL, triiodothyronine (T3) 89 ng/dL), as were calcium (9 mg/dL) and parathyroid hormone levels (35 pg/mL). Fine-needle aspiration biopsy demonstrated cytological features consistent with papillary thyroid carcinoma. She was admitted to the oncology surgery service for a planned total thyroidectomy. During the preoperative evaluation, heart sounds were noted to be best heard in the right hemithorax. An electrocardiogram revealed right axis deviation with inverted P waves in leads I, aVR, and aVL (Figure 1).

**Figure 2:**
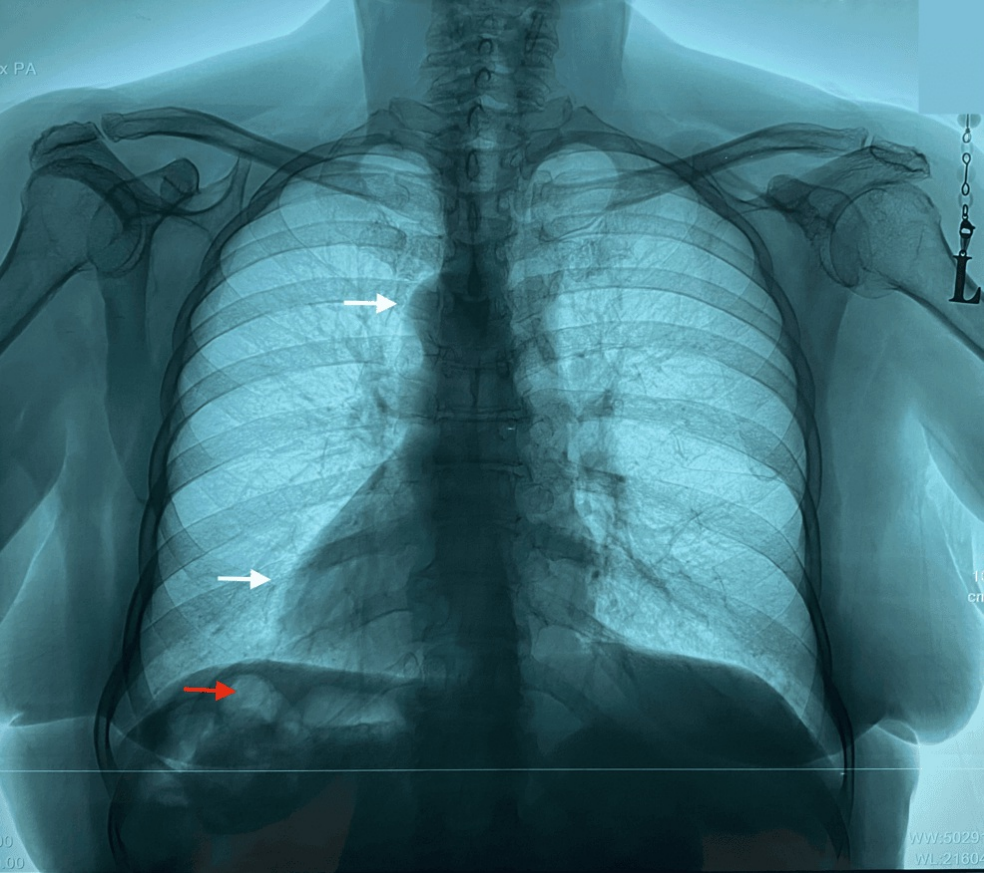
Chest radiograph showing a right-sided aortic knuckle and dextrocardia (white arrows), along with a right-sided gastric bubble (red arrow).

Subsequent posteroanterior chest radiograph demonstrated the cardiac apex positioned within the right hemithorax, confirming dextrocardia. The mediastinal silhouette revealed a right-sided projection of the aortic arch (aortic knuckle) along with a left-sided ascending aorta (Figure 2). These findings prompted further imaging to assess for associated visceral anomalies.

**Figure 3:**
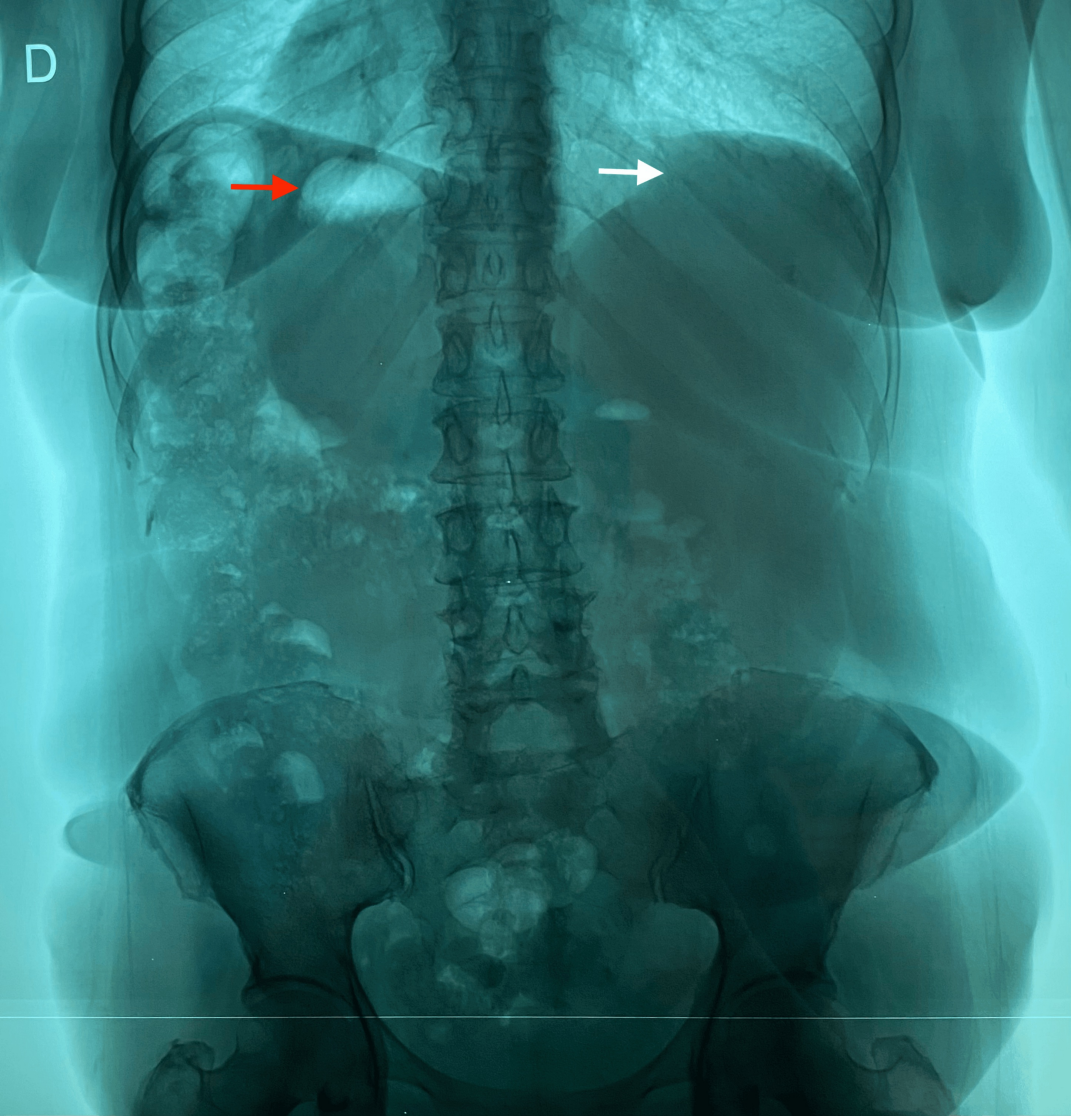
Abdominal radiograph demonstrating a left-sided hepatic shadow (white arrow) and the gastric bubble in the right upper quadrant (red arrow).

Additionally, an upright abdominal radiograph demonstrated a soft tissue density in the left upper quadrant consistent with the hepatic shadow, and a gas-filled gastric bubble projected in the right upper quadrant (Figure 3). This reversal of the normal anatomical orientation of the upper abdominal viscera further supported the suspicion of a mirror-image visceral arrangement, characteristic of situs inversus.

Abdominal ultrasound demonstrated a mirror-image transposition of the abdominal organs without abnormalities. Echocardiography revealed no structural cardiac defects. A diagnosis of SIT was made. The patient underwent an uncomplicated total thyroidectomy. Histopathology showed multiple foci in both lobes and the isthmus, the largest measuring 3 × 2 × 1.5 cm, with capsular invasion and extension into prethyroidal tissues and skeletal muscle. Postoperative calcium and thyroid function remained stable, and the patient was discharged on day 2 with levothyroxine. Follow-up included monitoring of calcium and thyroid hormone levels, neck ultrasound for recurrence, and consideration of radioactive iodine therapy.

## Discussion

This case describes a 60-year-old female patient with papillary thyroid carcinoma, in whom SIT was incidentally diagnosed during preoperative evaluation. To the best of our knowledge, only two cases have been previously reported documenting the coexistence of these two conditions. Chen et al. reported an 83-year-old woman with papillary thyroid carcinoma in whom SIT was incidentally confirmed by a thallium-201 scan [7]. Similarly, Charyshkin et al. described a 70-year-old man with lung metastases from papillary thyroid carcinoma, where thoracic and abdominal computed tomography confirmed both the lung tumor and the diagnosis of SIT [8]. Several reports have documented the coexistence of SIT with various malignancies, including breast, esophageal, cervical, and lung cancers [5,9]. However, the nature of this association remains unclear, and further epidemiological studies are needed to elucidate any potential link [5].

SIT is a rare congenital condition with an estimated incidence of 1 in 10,000 live births and is typically inherited in an autosomal recessive pattern [1]. Although the precise etiology remains unclear, evidence suggests that disruptions in left-sided determinants of gut rotation may impair normal gastrointestinal development, leading to a mirror-image configuration. This process is believed to involve alterations in key signaling pathways, including NODAL, LEFTY, and PITX2 [10,11].

Beyond descriptive reporting, it is also important to consider embryological and genetic perspectives in SIT coexisting with malignancy. While a causal link to thyroid carcinoma is unproven, advanced imaging in SIT patients has occasionally revealed congenital cardiovascular anomalies that share embryological pathways with thyroid and foregut development, raising the possibility of subtle developmental overlaps [11]. Genes such as NODAL and PITX2, implicated in left-right patterning, may influence not only visceral situs but also susceptibility to abnormal cellular growth, though this remains speculative.

In patients with dextrocardia, such as in our case, standard ECG lead placement produces characteristic changes that reflect the mirror-image orientation of the heart. Typical findings include right-axis deviation, inversion of P and T waves in lead I, a positive QRS in aVR, and poor R-wave progression in the precordial leads. These changes occur due to aberrant recording of septal depolarization and the altered orientation of cardiac chambers, whereas the inferior leads remain largely unaffected because of normal depolarization and orientation in the superior-inferior axis [12]. Accurate electrocardiographic assessment requires reversal of limb leads and mirrored precordial lead placement to reflect the true cardiac orientation. Recognition of these findings is clinically important, as misinterpretation of conventional ECGs can obscure ischemic changes or other pathologies. In our patient, the observed right-axis deviation and inverted P waves in leads I, aVR, and aVL were consistent with dextrocardia and were essential in prompting further imaging, ultimately confirming SIT [12,13].

The incidental diagnosis of SIT in this patient underscores the critical role of preoperative imaging in avoiding misinterpretation of anatomical landmarks during surgery. In a retrospective analysis, Eitler et al. highlighted that patients with SIT undergoing abdominal or thoracic procedures face elevated perioperative risks due to their mirror-image anatomy, thus requiring meticulous preoperative planning to minimize surgical complications [1]. In the present case, no intraoperative challenges were encountered, likely due to comprehensive preoperative evaluation and the absence of associated anatomical abnormalities.

Anesthetic management is equally critical. Reports from 2024 describe challenges in airway management, vascular access, and ECG monitoring in SIT patients undergoing abdominal cancer surgery, emphasizing that anesthesiologists must anticipate reversed anatomy for intubation, central venous catheterization, and intraoperative monitoring [14,15]. For thyroid surgery, these principles remain applicable, particularly when neck vascular structures follow mirrored orientations. Preoperative multidisciplinary planning should therefore extend beyond surgical considerations to encompass anesthesia and emergency preparedness.

Comparisons with other SIT-associated malignancies, such as colorectal and gastric cancers, provide further context. Recent case reports demonstrated that laparoscopic and robotic resections in SIT required modifications of surgeon positioning, trocar placement, and operative orientation. These cases underline that oncologic surgery in SIT, regardless of tumor site, demands tailored strategies to navigate mirror anatomy [16-18].

Although SIT is not inherently linked to an increased risk of malignancy, prior reports have noted that reversed organ positioning can pose diagnostic challenges and complicate recognition of certain tumor types [5,9]. This emphasizes the importance of recognizing SIT when interpreting imaging and planning surgical interventions, particularly in cases of intra-abdominal neoplasms. In our patient, imaging revealed complete thoracoabdominal transposition without structural or functional abnormalities, permitting a standard surgical approach despite the rarity of the anatomical configuration.

A recent population-based study investigating the relationship between situs anomalies and various abdominopelvic malignancies found no significant association between SIT and increased incidence of thyroid carcinoma, suggesting that such coexistence may be incidental rather than causative [19]. However, the authors noted that unrecognized SIT may lead to delays in diagnosis due to atypical anatomical presentations. In our case, early identification of SIT during the preoperative workup enabled timely management, further reinforcing the value of routine imaging, even in asymptomatic individuals.

## Conclusions

In conclusion, this report describes an unusual coexistence of SIT and papillary thyroid carcinoma, a combination rarely documented in the literature. While no causal link can be established from a single case, our findings underscore the importance of routine preoperative imaging and careful multidisciplinary planning when unexpected anatomic variants are identified. Awareness of SIT is critical to avoid diagnostic delays, prevent intraoperative complications, and optimize anesthetic and surgical strategies. Furthermore, this case highlights the need for larger studies to explore potential associations between SIT and malignancies and to better define perioperative management strategies in this unique patient population.
